# Molecular cloning and characterization of a thioredoxin from *Taiwanofungus camphorata*

**DOI:** 10.1186/s40529-014-0077-z

**Published:** 2014-12-04

**Authors:** Yu-Ting Chen, Pin-Feng Hong, Lisa Wen, Chi-Tsai Lin

**Affiliations:** 1 Department of Bioscience and Biotechnology and Center of Excellence for the Oceans, National Taiwan Ocean University, 2 Pei-Ning Rd, Keelung, 202 Taiwan; 2 Institute of Genomics and Bioinformatics, Agricultural Biotechnology Center, National Chung Hsing University, Taichung, Taiwan; 3 Department of Chemistry, Western Illinois University, 1 University Circle, Macomb, 61455-1390 IL USA

**Keywords:** Taiwanofungus camphorata, Thioredoxin (Trx), Three-dimension structural model, Insulin

## Abstract

**Background:**

Thioredoxin (Trx) is reduced by thioredoxin reductase. Trx is used in ribonucleoide reduction, assimilatory sulfate reduction, in modulation of protein sulfhydryl groups, and refolding proteins.

**Results:**

A TcTrx (Tc: *Taiwanofungus camphorata*) cDNA (640 bp, GenBank AY838902.1) encoding a putative thioredoxin (Trx) of 135 amino acid residues with calculated molecular mass of 16.17 kDa was cloned from *Taiwanofungus* ***c*** *amphorata*. The deduced amino acid sequence containing a motif (Cys-Gly-Pro-Cys) that is highly conserved among the reported Trxs. A three dimensional structural model of the TcTrx has been created based on the known structure of *Malassezia sympodialis* Trx (MsTrx, PDB ID: 2j23). To characterize the TcTrx, the codon optimized coding region was subcloned into an expression vector and transformed into *Saccharomyces cerevisiae*. The recombinant His8-tagged TcTrx was expressed and purified by Ni affinity chromatography. The purified enzyme showed a band of approximately 32 kDa (expected dimeric form) on a 12% SDS-PAGE. The molecular mass determined by MALDI-TOF is 33.16 kDa which suggests that the purified enzyme is a dimeric enzyme. Furthermore, the enzyme exhibited TcTrx activity via insulin assay. The Michaelis constant (*K*_*M*_) value for insulin was 3.78 × 10^−2^ mM. The enzyme’s half-life of deactivation was 13 min at 45°C. The enzyme was most active at pH 7.

**Conclusions:**

A three dimensional structural model of *T. camphorata* Trx based on its TcTrx cDNA sequence. The active form of the TcTrx has been successfully expressed in yeast. The enzyme possesses Trx activity and is capable of reduction of disulfide bonds during the formation of newly synthesized proteins.

**Electronic supplementary material:**

The online version of this article (doi:10.1186/s40529-014-0077-z) contains supplementary material, which is available to authorized users.

## Background

*Taiwanofungus camphorata* (*T. camphorata*) is a valued mushroom found only in the forests of Taiwan. It has been used for centuries as health food, among others (Ao et al. [2009]). *T. camphorata* has been shown to have anti-inflammatory properties (Hsieh et al. [2010]). The active compounds identified from the fruiting bodies of *T. camphorata* in a submerged culture are benzenoids, diterpenes, maleic and succinic acid derivatives, polysaccharides, steroids, and triterpenoids (Ao et al. [2009]). It can be obtained as health supplements formulated from the mass of *T. camphorata* from the artificial cultivation by an Asian Nova Biotechnology Inc (http://www.asian-bio.com/) at a high market value. Many studies were aimed to find the exact bioactive compounds of the mushroom (Ao et al. [2009]). In order to search for physiologically active components including antioxidant enzymes, we have established an expressed sequence tag (EST) from the fruiting bodies of *T. camphorata.* Based on the established EST*,* several antioxidant enzymes including a 2-Cys peroxiredoxin (Huang et al. [2007]), a catalase (Ken et al. [2008]), a dithiol glutaredoxin (Ken et al. [2009]), a 2-Cys peroxiredoxin isozyme (Liau et al. [2010]), a monothiol glutaredoxin (Ken et al. [2011]), a nitroreductase (Chen et al. [2012]), a peroxiredoxin (Huang et al. [2014]) and an aryl-alcohol dehydrogenase (Ken et al. [2014]) have been cloned and characterized. This encourages us further to search for active components from the established EST of *T. camphorata* for potential health food applications.

Thioredoxin (Trx) is reduced by thioredoxin reductase with NADPH as the hydrogen donor. Trx is used in refolding proteins ((Li and Churchich [1997]), in modulation of protein sulfhydryl groups (Buchanan et al. [1994]), and phage assembly (Russel and Mode [1986]). Here, we report a three dimensional structural model of *Taiwanofungus camphorata* thioredoxin based on its sequence. The coding sequence of the TcTrx cDNA was introduced into an *S. cerevisiae* expression system and the active enzyme purified and characterized its properties.

## Methods

### Isolation of TcTrx cDNA

We have previously established an EST database from fruiting bodies of *T. camphorata* and sequenced all clones with insert size greater than 0.4 kb (data not shown). The identity of a Trx cDNA clone was assigned by comparing the inferred amino acid sequence in various databases using the basic local alignment search tool (BLAST) (http://www.ncbi.nlm.nih.gov/blast/Blast.cgi). The Trx cDNA fragment was subcloned into pCR®4-TOPO® (invitrogen, CA) vector and transformed into *E. coli* TOPO10. The nucleotide sequence of the insert was determined in both strands. Sequence analysis revealed that the Trx cDNA covered an open reading frame of a putative Trx cDNA (640 bp, GenBank AY838902.1).

### Bioinformatics analysis of TcTrx

The BLAST program was used to search homologous protein sequences in the nonredundant database (NRDB) at the National Center for Biotechnology Information, National Institutes of Health (http://www.ncbi.nlm.nih.gov/). Multiple alignments were constructed using ClustalW2 program. Protein secondary structure was predicted by SWISS-MODEL program and represented as α helices and β strands. A three dimensional structural model of TcTrx was created by SWISS-MODEL (Arnold et al. [2006]) (http://swissmodel.expasy.org/) based on the known crystal structure of *Malassezia sympodialis* Trx (MsTrx, PDB ID: 2j23).

### Subcloning of TcTrx cDNA into an expression vector

**Figure 1:**
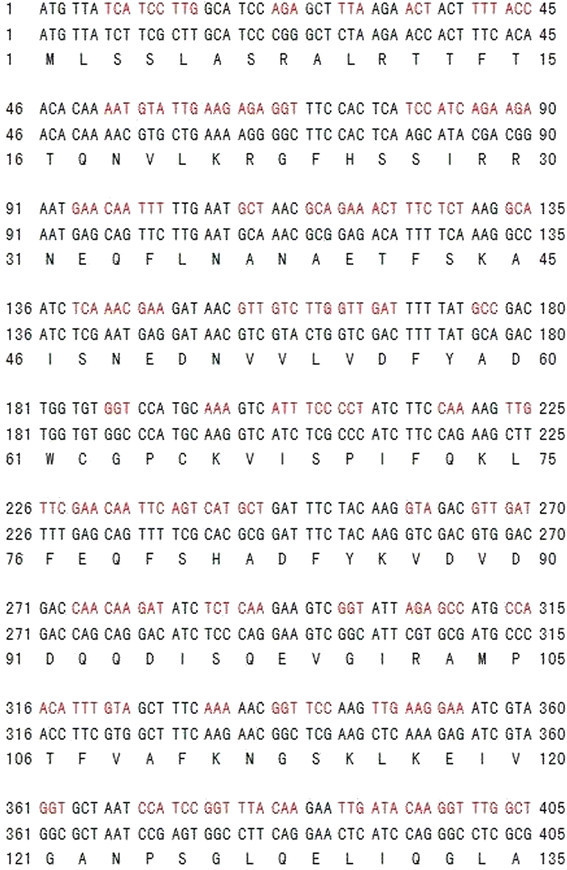
**Nucleotide sequences of TcTrx cDNA and its codon usage optimization based on the yeast codon usage table.** The codon optimization of TcTrx was shown above with red codon.

TcTrx cDNA was subcloned into an *E. coli* and yeast expression vector, respectively. The coding region of the TcTrx cDNA was amplified using gene specific flanking primers. The 5′ upstream primer contains *Eco* RI recognition site (5′*GAA TTC* GAT GTT ATC TTC GCT TGC ATC C3′) and the 3′ downstream primer contains *Eco* RI recognition site (5′*GAA TTC* GCG AGG CCC TGG ATG AG3′). Using 0.2 μg of TcTrx cDNA as a template, and 10 pmole of each 5′ upstream and 3′ downstream primers, a 405 bp fragment encoding the putative mature TcTrx gene was amplified by PCR. The fragment was ligated into pCR^Ò^4-TOPO^Ò^ and transformed into *E. coli*. The recombinant plasmid was isolated and digested with *Eco* RI. The digestion products were separated on a 1.0% agarose gel. The 405 bp insert DNA was gel purified and subcloned into *Eco* RI site of pET-20b(+) expression vector (Novagen, Darmstadt, Germany). The recombinant DNA was then transformed into *E. coli* C43(DE3). However, the recombinant protein was not expressed in the *E. coli* expression system. We then subcloned the gene into the *Eco* RI site of the pYEX-S1 expression vector (Clontech, Mountain View, CA, USA) and introduced into *Saccharomyces cerevisiae* (trp^−^ ura^−^), The recombinant protein was still not expressed in the *Saccharomyces cerevisiae* expression system. We decided to optimize the TcTrx DNA sequenced based on the yeast codon usage table (The codons were optimized by using the Codon Optimization Tool provided by the Integrated DNA Technologies (http://sg.idtdna.com/CodonOpt) with codon usage table of *Saccharomyces cerevisiae.* The GenScript Codon Usage Frequency Table Tool was used as the reference for yeast usage table. The optimized gene was custom synthesized by Genomics company, Taiwan. The optimized sequence is shown in Figure 1 in red. It was subcloned into a pET-20b(+) expression vector and the recombinant DNA transformed into *E. coli* C43(DE3). The recombinant protein was still not expressed in the *E. coli* expression system. We then re-amplified the codon-optimized pET-20b(+)-TcTrx DNA using two gene-specific primers: the 5′ upstream primer contained *Eco* RI recognition site (5′*GAA TTC* GAT GTT ATC TTC GCT TGC ATC C3′) and the 3′ downstream primer contained a His8-tag and *Eco* RI recognition site (5′ *GAATTC* GAG ACG TCA GTG GTG GTG GTG GTG GTG GTG GTG3′). Using the 0.2 μg optimized recombinant DNA of pET-20b(+)-TcTrx as a template, and 10 pmole of each 5′ upstream and 3′ downstream primers, a 0.4 kb fragment was amplified by PCR. The fragment was ligated into pCR^Ò^4-TOPO^Ò^ and transformed into *E. coli*. The recombinant plasmid was isolated and digested with *Eco* RI. The digestion products were separated on 1.0% agarose gel. The 0.4 kb insert DNA was gel purified and subcloned into the *Eco* RI site of the pYEX-S1 expression vector and introduced into *Saccharomyces cerevisiae* (trp^−^ ura^−^). The transformed yeast cells were selected by YNBDT (0.17% yeast nitrogen base, 0.5% ammonium sulfate, and 2% glucose) agar plates containing 20 μg Trp/mL. The presence of TcTrx cDNA in the selected transformants was verified by PCR using gene-specific flanking primers. The recombinant TcTrx protein was expressed in yeast in YPD medium (1% yeast extract, 2% peptone, 2% glucose). Expression of the functional recombinant TcTrx was analyzed by enzyme activity assay.

### Expression and purification of the recombinant TcTrx

The yeast transformant containing the TcTrx gene was grown at 30°C, 170 rpm in 100 mL of YPD medium for 18 h. The cells were harvested and the soluble proteins extracted in PBS (phosphate buffer saline) with glass beads as described previously (Ken et al. [2005]). The recombinant TcTrx was purified by Ni-NTA affinity chromatography (elution buffer: 1× PBS/5% glycerol/1 mM DTT containing 20–250 mM imidazole) according to manufacturer’s instruction (Qiagen). The purified protein was analyzed by a 12% SDS-PAGE followed by staining with Coomassie Brilliant Blue R-250 and destaining with 10% acetic acid/10% methanol. The purified protein was pooled, desalted, and buffer exchanged using Amicon ultra centrifugal filter unit (5000 MWCO). The exchanged buffer exchanged was 0.01 × PBS/0.01 mM DTT/2.5 mM imidazole/0.1% glycerol. The final recombinant TcTrx protein concentration was determined by a Bio-Rad Protein Assay Kit (Richmond, CA). For storage, one volume of glycerol was added to the purified protein and stored at −20°C for further analysis.

### Molecular mass analysis via JOEL MALDI-TOF

The purified recombinant TcTrx (0.82 μg/μL) was prepared in 0.01 × PBS containing 0.01 mM DTT, 2.5 mM imidazole and 0.1% glycerol. The sample (5 μL) was used for molecular mass determination using an MALDI-TOF mass spectrometer (JMS-S3000, Japan).

### TcTrx activity assay

Trx activity was assayed by the method of Holmgren (Holmgren and Reichard [1967]; Holmgren [1979a], [b]) using the insulin precipitation assay which was monitored by a spectrophotometric assay of the increase in turbidity at 650 nm. The reaction mixture (200 μL) at 25°C contained 0.1 M potassium phosphate (pH 7.0), 2 mM EDTA, 0.33 mM DTT, 15 mM NADPH, 0.025 mM insulin and 1.0 μg TcTrxR (thioredoxin reductase from *T. camphorata*, Huang et al. [2010]). The reaction was started by the addition of 1.0 μg TcTrx (0.41 μg/μL). The reaction was followed by the increase in *A*_*650*_ due to insulin precipitation on reduction.

### Kinetic studies

The kinetic properties of the TcTrx (1.0 μg) was determined by varying the concentrations of insulin (0.025 ~ 0.055 mM). The change in absorbance at 650 nm was recorded between 30 and 60 sec. The *K*_M_, V_max_ and *k*_cat_ were calculated from Lineweaver-Burk plots.

### Enzyme characterization

The TcTrx enzyme was tested for stability in terms of its activity under various conditions. Aliquots of the TcTrx sample (1.0 μg) were treated as follows: (1) *Thermal effect*. Each enzyme sample (1.0 μg) was heated at 40, 50, or 60°C for 5 min. Then samples were checked for TcTrx activity: 80% residual activity at 40°C treatment, 40% residual activity at 50°C treatment. Therefore, we choose at 45°C heating to this enzyme effect, each enzyme sample (1.0 μg) was heated at 45°C for 2, 4, 8, 16 min. (2) *pH effect*. Each enzyme sample (1.0 μg) was adjusted to desired pH by adding a half volume of buffer with different pHs: 0.2 M citrate buffer (pH 4.0), 0.2 M phosphate buffer (pH 6.0, 7.0 or 8.0) or 0.2 M CAPS buffer (pH 10.0). Each sample was incubated at 37°C for 30 min. At the end of each treatment, samples were checked for TcTrx activity by insulin precipitation assay at pH 7.

## Results and discussion

### A three dimensional structural model of *Taiwanofungus camphorata* thioredoxin based on its TcTrx cDNA sequence

**Figure 2:**
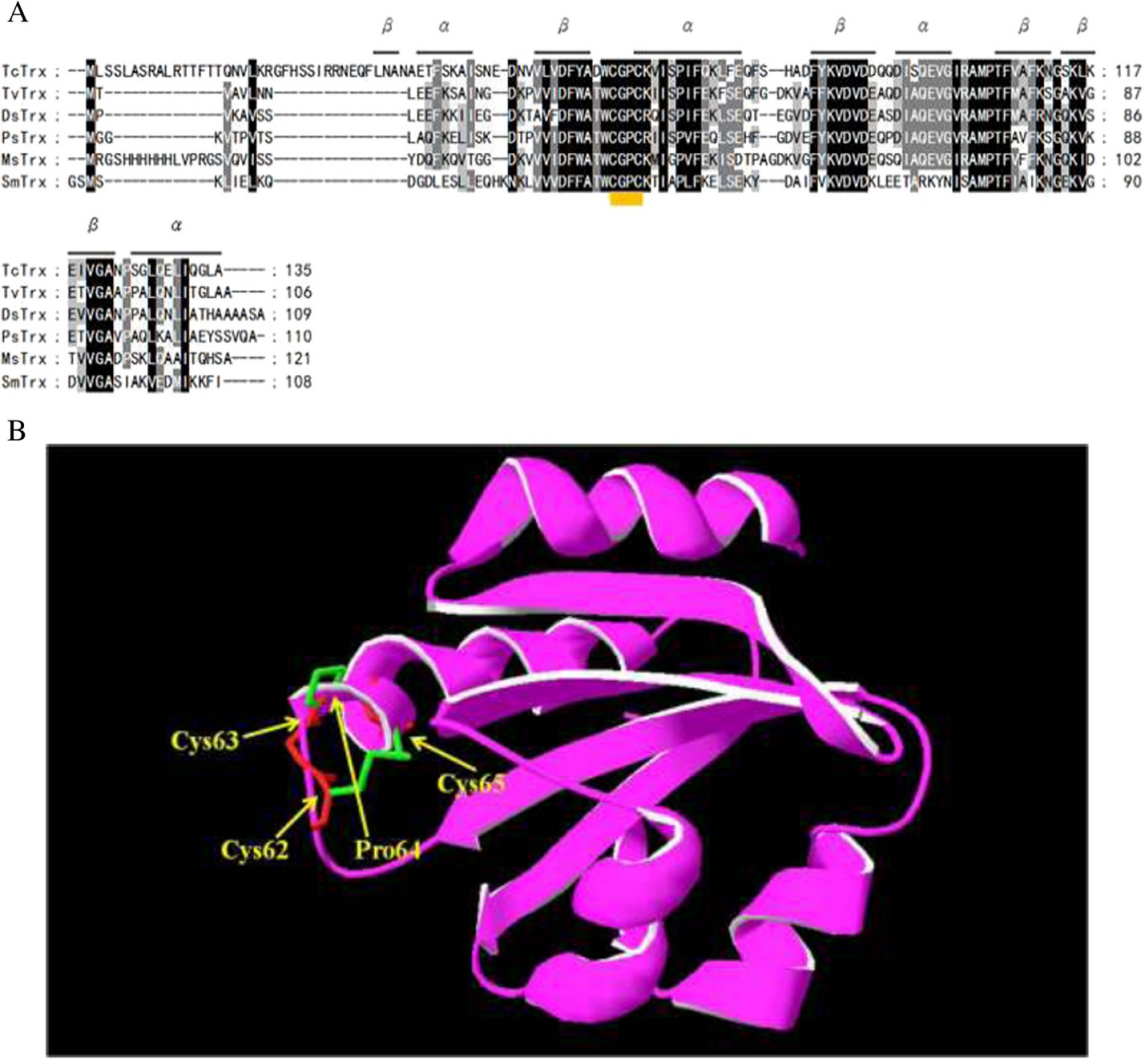
**Alignment of the amino acid sequences of TcTrx with other organism’s Trx and three dimensional structural model. (A)** Sequence alignment: TcTrx (this study), TvTrx (*Trametes versicolor,* EIW63845.1), DsTrx (*Dichomitus squalens,* EJF67406.1), PsTrx (*Punctularia strigosozonata,* EIN14269.1), MsTrx (*Malassezia sympodialis,* AJ937746.1), SmTrx (*Schistosoma mansoni,* 2XBI_A). Identical amino acids in all sequences are shaded black, conservative replacements are shaded gray. The highly conserved motif is denoted in yellow underline (Figure 1A) predicted by SWISS-MODEL program and represented as α helices and β strands. **(B)** A three dimensional structural model of TcTrx. The structural model of the TcTrx was created based on the known crystal structure of *Malassezia sympodialis* (MsTrx, PDB ID: 2j23) via SWISS-MODEL program. Superimposition of TcTrx (purple) and SmTrx (white) was shown using protein solid ribbons. Yellow structure denotes active site.

A putative TcTrx cDNA (640 bp) clone was identified on the basis of the consensus pattern and sequence homology to other published Trxs in NCBI database. The entire coding region of TcTrx cDNA is 405 bp and the deduced protein consists of 135 amino acid residues with a calculated molecular mass of 16.2 kDa (accession no. GenBank AY838902.1). The TcTrx gene was optimized based on yeast codon usage table as shown Figure 1 red color codon. Figure 2 shows the optimal alignment of the amino acid sequences of the TcTrx with five selected Trx sequences from other sources. This TcTrx shared 63% similarity with TvTrx (*Trametes versicolor*, accession no. EIW63845.1), 62% similarity with DsTrx (*Dichomitus squalens*, accession no. EJF67406.1), 58% similarity with PsTrx (*Punctularia strigosozonata*, accession no. EIN14269.1), 57% similarity with MsTrx (*Malassezia sympodialis*, accession no. AJ937746.1, PDB ID: 2j23), and 42% similarity with SmTrx (*Schistosoma mansoni*, accession no. 2XBI_A). The deduced amino acid sequence containing highly conserved active site motif (Cys^62^-Gly-Pro-Cys^65^) (Limacher, et al. [2007]). The two Cys residues are the key to the ability of thioredoxin to reduce other proteins. A three dimensional structural model of the TcTrx (purple) has been created based on the known structure of *Malassezia sympodialis* Trx (MsTrx, PDB ID: 2j23). The RMSD is 0.95 Å. The highly conserved active motif is denoted in yellow underline (Figure 2A). The secondary structure, predicted by SWISS-MODEL program, showed 4 α helices and 6 β strands. Superimposition with SmTrx (PDB ID: 2XBI, white) was shown using protein solid ribbons. Putative active residues are shown in yellow (Figure 2B).

### Expression and purification of the recombinant optimized TcTrx

**Figure 3:**
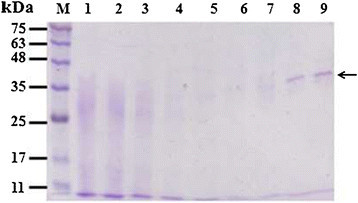
**Expression and purification of recombinant TcTrx in** ***Saccharomyces cerevisiae.*** Fifteen μL (loading buffer without mercaptoethanol and without boiling) of each fraction was loaded into each lane of the 12% SDS-PAGE followed by Coomassie Brilliant Blue R-250 staining. Lane 1, crude extract from *Saccharomyces cerevisiae* expressing TcTrx; 2, flow-through proteins from the Ni-NTA column; 3, wash; 4–9, TcTrx eluted from Ni-NTA column. Molecular masses (in kDa) of standards are shown at left.

The optimized coding region for the TcTrx (405 bp) was amplified by PCR and subcloned into an expression vector, pYEX-S1 as described in the Materials and Methods. Positive clones were verified by DNA sequence analysis. The recombinant TcTrx was expressed, and the proteins were analyzed by a 12% SDS-PAGE in the absence of reducing agent and without boiling (Figure 3). The recombinant TcTrx was expressed as a His_8_-tagged fusion protein and was purified by affinity chromatography with nickel chelating Sepharose. A major band with molecular mass of ~35 kDa (expected size of TcTrx dimer) was detected in Ni-NTA eluted fractions by SDS-PAGE (Figure 3, lanes 8–9). Analysis of the TcTrx by MALDI-TOF MS confirms the presence of a single protein with molecular mass of 33154 Da. This indicates that the enzyme is predominantly dimeric in nature. As shown in Figure 2, from the point of the structure that Cys62 and Cys65, we assume that the Trx favors formation of dimers through intermolecular disulfide linkages. This assumption is supported by our SDS-PAGE analysis of the purified protein in the absence of β-mercaptoethanol, the dimeric form is predominant (see Figure 3, lanes 8–9). The dimers may be linked by two disulfide bonds between Cys62-Cys65 and Cys65-Cys62, or between Cys62-Cys62 and Cys65-Cys65. The dimers may also be linked by one disulphide bond between Cys62 and Cys65, or Cys62-Cys62, or Cys65-Cys65 (Chae et al. [1994]; Liau et al. [2010]). The yield of the purified His_8_-tagged TcTrx was 82 μg from 100 mL of culture. Functional TcTrx was detected by activity assay as describe below.

### Kinetic studies of the purified TcTrx

**Figure 4:**
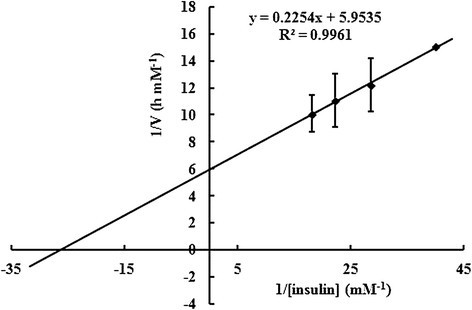
**Double-reciprocal plot of varying insulin on TcTrx activity.** The initial rate of the enzymatic reaction was measured with change in absorbance at 650 nm between 30 and 60 sec with the insulin varied from 0.025 ~ 0.055 mM. The *K*_M_*, k*_cat_ and *k*_cat_/*K*_M_ were calculated from Lineweaver-Burk plots.

As shown in Figure 4, the Lineweaver-Burk plot of the velocity (1/V) against 1/[insulin] gave the *K*_M_*, k*_cat_ and *k*_cat_/*K*_M_ values were 3.7×10^−2^ mM, 1.2×10^−2^ min^−1^, 3.1×10^1^ min^−1^ mM^−1^. Comparison of the *K*_M_ with that of Trx from other sources (Table 1) reveals that *T. camphorata*’s *K*_M_ is several fold larger. The result indicating that the TcTrx works at higher substrate concentration. But the *k*_cat_ value is similar to that of Trx from other sources.

**Table 1:** **Kinetic characterization of TcTrx and that from other sources**

Protein	Substrate	***K***_m_(mM)	***k***_cat_	***k***_cat_/***K***_m_
TcTrx	insulin	3.7 × 10^−2^	1.2	3.1 × 10^1^
*E. coli* Trx	insulin	1.1 × 10^−2^		
Dv Trx	insulin	6.3 × 10^−3^		
Dr Trx	insulin	5.7 × 10^−3^	1.1	1.9 × 10^2^

Values are from TcTrx (this study), *Escherichia coli* Trx (*E. coli* Trx) (Holmgren [1979a]), *Desulfovibrio vulgaris* Trx (Dv Trx) (Pieulle et al. [2011]) and *Deinococcus radiodurans* Trx (Dr Trx) (Obiero et al. [2010]).

### Properties of the purified TcTrx

**Figure 5:**
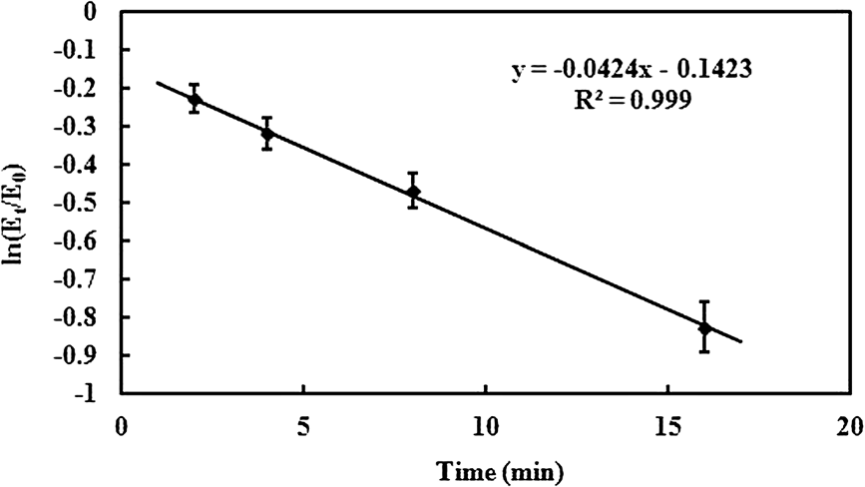
**Effect of temperature on the purified TcTrx.** The enzyme sample (1.0 μg) was heated at 45°C for 2, 4, 8, 16 min. At the end of each treatment, samples were assayed for TcTrx activity at pH 7.0.

**Figure 6:**
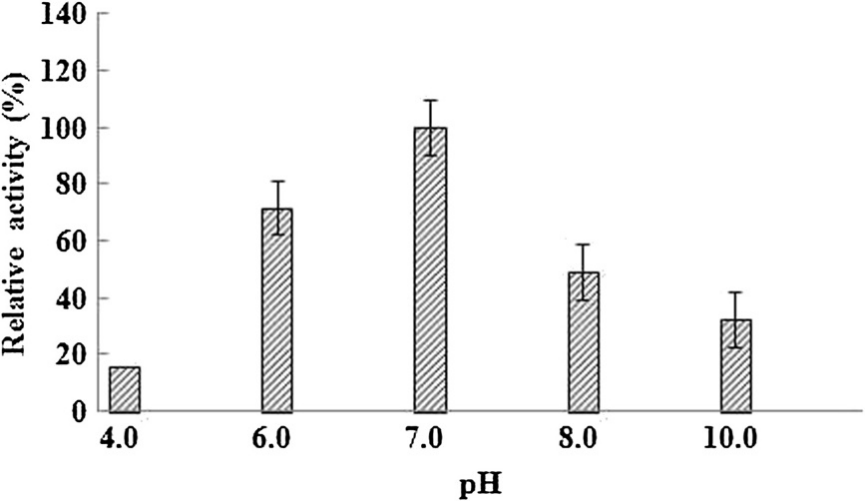
**Effect of pH on the purified TcTrx.** Aliqouts of the enzyme sample were incubated with different pH buffers at 37°C for 30 min and then assayed for TcTrx activity. Data are means of three experiments.

The TcTrx enzyme was shown to possess Trx activity by its ability to reduction insulin. Heat stability of the TcTrx was tested to examine the effect of heat on the Trx activity as described in the Materials and Methods. We found 80% and 40% residual activity when the enzyme was treated at 40°C and 50°C for 5 min, respectively. The enzyme activity found at 25°C was defined as 100% activity. The enzyme (1.0 μg) was further heated for various time intervals at 45°C. The enzyme activity decreased as the heating time increased (Figure 5). The enzyme’s half-life of deactivation was 13 min at 45°C. In Figure 6, the TcTrx is most activity under pH 7.0.

## Conclusion

A three dimensional structural model of *T. camphorata* Trx based on its TcTrx cDNA sequence. This study reported the first cloning and expression of an important reduction enzyme, TcTrx, from *T. camphorata*. The active form of the TcTrx has been successfully expressed in yeast. The enzyme possesses Trx activity and is capable of reduction of disulfide bonds during the formation of newly synthesized proteins.

The TcTrx has a higher *K*_M_ value (Table 1) therefore can work under higher substrate concentration. Its *k*_cat_ value is compatible to that of Trx from another source. It is likely that the Trx is one of the enzymes responsible for reducing protein disulfide targets in *T. camphorata*.

## Authors’ original submitted files for images

Below are the links to the authors’ original submitted files for images. Authors’ original file for figure 1 Authors’ original file for figure 2 Authors’ original file for figure 3 Authors’ original file for figure 4 Authors’ original file for figure 5 Authors’ original file for figure 6

## Abbreviations

Trx: Thioredoxin
IPTG: Isopropyl β-D-thiogalactopyranoside
SDS-PAGE: Sodium dodecyl sulfate-polyacrylamide gel electrophoresis
PBS: Phosphate buffer saline
