# Supplementary figures and images for: Molecular cloning and characterization of a thioredoxin from Taiwanofungus camphorata
Source: Bot Stud. 2014 Dec 4;55:77. doi: 10.1186/s40529-014-0077-z (PMC5432747; doi:10.1186/s40529-014-0077-z)

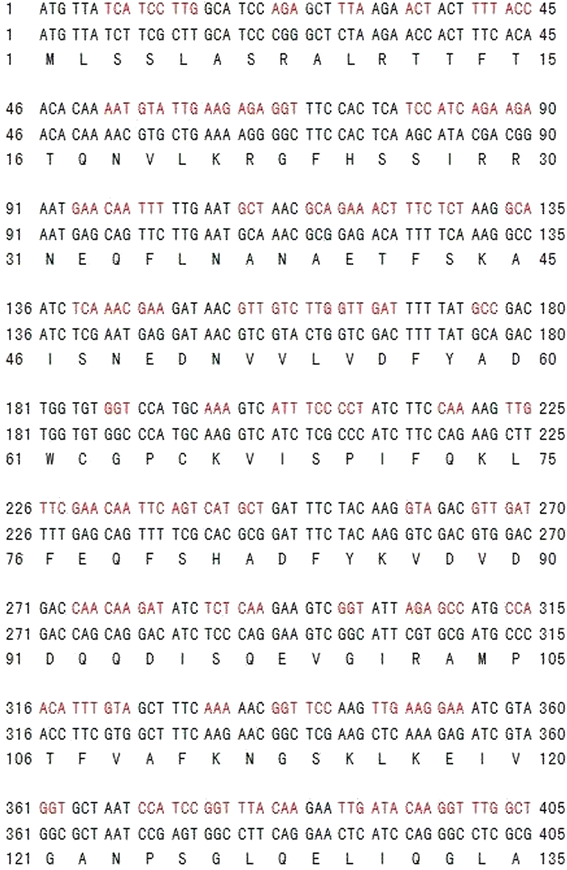

Supplement: Supplementary file 1 — Authors’ original file for figure 1 [file 40529_2014_9077_MOESM1_ESM.gif]

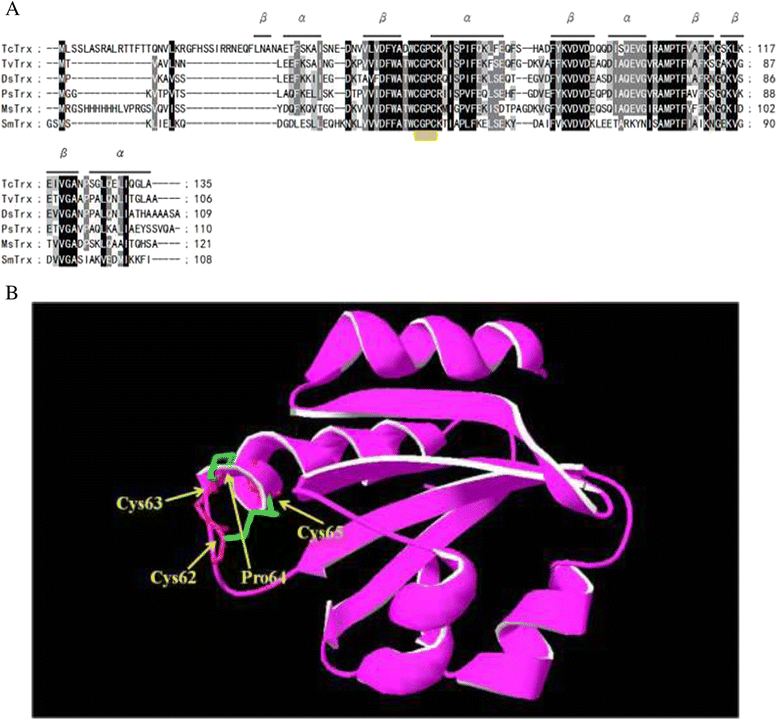

Supplement: Supplementary file 2 — Authors’ original file for figure 2 [file 40529_2014_9077_MOESM2_ESM.gif]

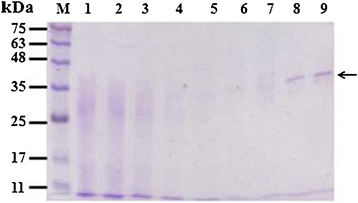

Supplement: Supplementary file 3 — Authors’ original file for figure 3 [file 40529_2014_9077_MOESM3_ESM.gif]

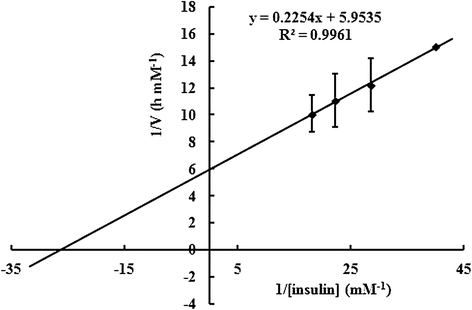

Supplement: Supplementary file 4 — Authors’ original file for figure 4 [file 40529_2014_9077_MOESM4_ESM.gif]

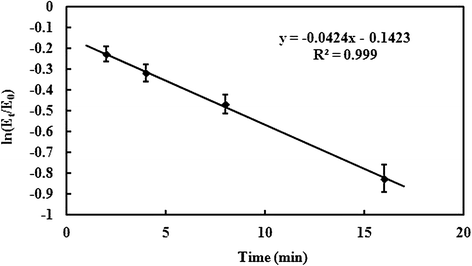

Supplement: Supplementary file 5 — Authors’ original file for figure 5 [file 40529_2014_9077_MOESM5_ESM.gif]

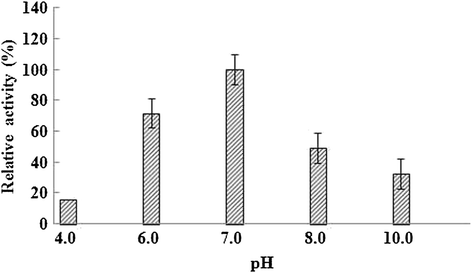

Supplement: Supplementary file 6 — Authors’ original file for figure 6 [file 40529_2014_9077_MOESM6_ESM.gif]
